# Comparative Evaluation of a New Tricalcium Silicate Cement With Calcium Hydroxide as Direct Pulp Capping Agents: A Clinical Study

**DOI:** 10.7759/cureus.56508

**Published:** 2024-03-19

**Authors:** Dhanya Shaji, Sobha Krishnamma, Divia Attuvalappil Rajan, Subija Kunnumpurath Narayanankutty, Jinu George, Christalin Ramakrishnan

**Affiliations:** 1 Department of Conservative Dentistry and Endodontics, Government Dental College, Thrissur, IND; 2 Department of Conservative Dentistry and Endodontics, Government Dental College, Kottayam, IND

**Keywords:** new tricalcium silicate cement, dycal, direct pulp capping, calcium hydroxide, biodentine

## Abstract

Background and objectives

Direct pulp capping (dPC) is a therapeutic process that involves the application of a protective chemical to an exposed pulp with the intent to facilitate the restoration and preservation of its vitality and function. Despite numerous proposed solutions, researchers have yet to find a dependable, non-absorbable bioactive pulp capping substance that constantly activates cellular healing processes, consequently preserving pulpal vitality over an extended period of time. The objective of this study was to assess and contrast the efficacy of a novel tricalcium silicate cement and calcium hydroxide in preserving the long-term health of the dental pulp following dPC using clinical and radiographic observations.

Materials and methods

A total of 60 individuals with symptoms of reversible pulpitis associated with deep carious lesions were chosen for dPC in the study. Two groups comprising 30 patients each underwent dPC using the novel tricalcium silicate cement (experimental group) and calcium hydroxide (control group) out of the total number of patients. A thin protective covering of self-curing glass ionomer base was applied on top of the capping material. The dentist provided instant permanent restoration employing direct posterior composite resin. Follow-up examinations were conducted three, six, nine, and 12 months after the procedure, during which assessments were performed using clinical and radiographic parameters.

Results

The minimum age of the study group was 16, and its maximum age was 34 (22.35 ± 2.3 years). The control group consisted of 12 males and 18 females, while the Biodentine group consisted of 13 males and 17 females. The age and gender distribution were determined to have insignificant statistical differences across the groups. The pain score exhibited statistical significance at both the three-month and six-month follow-up visits (p < 0.05). The pain score at the 12-month follow-up was 0.38 ± 0.52 in the case group and 0.42 ± 0.61 in the control group (p = 0.79). The average Periapical Index (PAI) score for the Dycal and Biodentin groups after the 12-month follow-up was 1.38 ± 0.97 and 1 ± 0.3, respectively. This difference was found to be statistically significant (p = 0.044). In addition, there was a statistically significant difference in the PAI score at the three-month, six-month, and nine-month follow-ups (p < 0.01). During the entirety of the follow-up duration, one individual in the Biodentine group had tenderness upon percussion, a negative reaction for pulp vitality, pulpal changes, and a widening of the periodontal space. Statistically significant differences were observed in pulpal changes and tenderness on percussion during the nine-month and 12-month follow-up periods (p < 0.05). After 12 months, the rate of success in the group treated with Dycal was 91.3%, while the success percentage in the group treated with Biodentine was 98.55%. This difference in outcomes was determined to be statistically significant (λ^2^ = 5.46; p = 0.019).

Conclusion

The study findings indicate that Biodentine, a novel tricalcium silicate cement, outperforms calcium hydroxide in preserving pulp vitality over the long term following dPC. The Biodentine group attained an overall success rate of 98.55%, whereas the Dycal group had a success rate of 91.3% following 12 months of subsequent follow-up visits.

## Introduction

Direct pulp capping (dPC) is a technique used in dentistry that involves placing cement directly on an exposed pulp to shield it from further damage. This allows the pulp to recover and heal itself while preserving its vitality. The objective is to delay the implementation of treatments that may ultimately diminish the overall prognosis for tooth preservation and functionality. Preserving the vitality of an exposed pulp is important for the long-term viability of the tooth, as the vital pulp has effective immune defense mechanisms [1]. dPC is regarded as suitable for treating permanent teeth that have been exposed to caries, trauma, or mechanical damage [2]. The success of vital pulp therapy relies extensively on the treatment approach, which involves removing the harmful stimulus, controlling infection, and isolating the dentin or pulp from further damage. It also includes using specific techniques to stimulate biological mechanisms that promote the growth of dentin and restore the cavity to prevent bacterial micro-leakage [3]. Ensuring the precise and secure insertion of a definitive repair during pulp capping is essential for achieving successful clinical outcomes [4].

The most reliable indicators for estimating survival rates in vital pulp therapy are clinical review and radiographic examination. It is crucial to emphasize the significance of follow-up following dPC since there may be instances of recurrent caries, restorative failure, a lack of oral hygiene, or additional concerns that require consideration [1]. The optimal material for dPC should possess the ability to manage infection, securely bond to dentin, inhibit the leaking of microorganisms, exhibit biocompatibility, provide easy manipulation, and eventually enable the establishment of a mineral-based tissue barrier. Various materials have been utilized for pulp capping; however, calcium hydroxide has commonly been employed and is considered the benchmark for pulp capping agents. Due to its capacity to promote the creation of hard tissue, it functions as a stimulant for the healing of pulpal tissue [3].

However, after applying this traditional alkaline substance for pulp capping, the nearby pulp tissue typically becomes severely disrupted and deformed, resulting in the formation of an obliteration region. The lack of adhesive properties in the cement and its gradual disintegration might result in micro-leaks and the infiltration of bacteria into the exposed area. Bacterial invasion can also happen through defects in calcium hydroxide-produced dentine bridges, known as “tunnel defects,” which may serve as a pathway for bacteria to migrate from the exposed region to the pulp [5]. The failure of pulp capping with calcium hydroxide can be attributed to various variables, including the obstruction of particles of the capping agent, the necrotizing activity of calcium hydroxide that affects the vascularity of the pulp up to depths of 1.5 mm, and disruption caused by a blood clot [3,6].

A novel bioactive cement called Biodentine has been introduced as a replacement for dentin. It possesses both the same indications and method of action as calcium hydroxide but lacks its drawbacks. The restorative cement is composed of calcium silicate and possesses mechanical properties comparable to dentin. It can be utilized as a replacement for dentin on crowns and roots, comparable to mineral trioxide aggregate (MTA). However, it offers various additional benefits, such as cost-effectiveness, enhanced handling features, and decreased setting time. It exerts a beneficial impact on vital pulp cells and promotes the development of tertiary dentin. Reparative dentin production is also facilitated when it comes into proximity with vital pulp [7]. This study aims to contribute to the existing literature on vital pulp therapy by investigating the efficacy of Biodentine and calcium hydroxide as dPC materials in preserving long-term pulp vitality.

## Materials and methods

The study was conducted at Government Dental College, Kottayam, and involved 60 patients exhibiting symptoms of reversible pulpitis with deep carious lesions in their permanent molars. These patients were selected from the outpatient Department of Conservative Dentistry and Endodontics at Government Dental College Kottayam under specific criteria for inclusion and exclusion.

The inclusion criteria comprised individuals aged 18 to 35 in good health without underlying medical conditions or medications. Patients exhibited indications of reversible pulpitis, evidenced by brief discomfort triggered by stimuli to the decayed tooth, which was alleviated post-removal. Positive responses to cold and electric pulp tests were observed, with no spontaneous pain or percussion-induced tenderness. Exclusion criteria included weakened immune systems, pregnancy at follow-up, tooth decay spread into pulp, signs of irreversible pulpitis, prolonged bleeding from exposure, and traumatic dental history.

The patients chosen were between the ages of 18 and 35. The study protocol received approval from the Institutional Ethics Committee with the ethical number IEC/M/03/2012/DCK. The sample size has been determined using the formula N = 2S2f(α,β)/d2. The variables in the equation are as follows: d represents the clinically significant difference, S represents the standard deviation, and N represents the sample size. The sample size necessary for the current investigation was determined to be 60 patients, with a margin of error of 5%. Due to the possibility of participants dropping out during the follow-up period, the sample size has been adjusted to 70 subjects.

The patients were allocated to each group using a process of random assignment utilizing a random number generator chart. The experimental group comprises the dPC process performed with the novel tricalcium silicate cement known as Biodentine. Calcium hydroxide (Dycal) was used for dPC in the control group. The study comprised permanent molars with severe carious lesions that were close to the pulp, and these molars were assessed using both clinical and radiographic strategies. The study participants consisted of individuals aged 18 to 35 who were in good health, without any underlying medical conditions, and not taking any medications.

All the chosen patients had indications of reversible pulpitis, characterized by brief episodes of discomfort that were triggered by stimuli to the decayed tooth and alleviated following its removal. Both the cold and electric pulp tests yielded positive results, and there was no previous evidence of spontaneous pain or percussion-induced tenderness. The Periapical Index (PAI) score was 1, suggesting that the study encompassed normal periradicular components. All the patients recruited for the study exhibited no significant responsiveness to finger pressure, no signs of deterioration in the pulp based on clinical or radiographic data, effective control of bleeding at the point of exposure within a time frame of no more than five minutes, a lack of any serous or purulent discharge from the exposed area, and no indication of non-carious damage or developmental abnormalities. The teeth that were not exposed to traumatic or abnormal functional pressures were considered. The following individuals were excluded from the study: patients with weakened immune systems, pregnant patients at the time of subsequent follow-up, patients with tooth decay that has spread into the pulp chamber, patients with teeth displaying indications of irreversible pulpitis, and patients experiencing bleeding from the point of exposure for longer than five minutes.

The materials utilized in this study included Biodentine, a novel tricalcium silicate cement employed for dPC in the experimental group, and calcium hydroxide (Dycal) utilized in the control group. Local anesthesia was administered using Lignocaine HCl 2% with 1:80,000 adrenaline for pain management during procedures. For caries removal, mechanical excavation was conducted using diamond burs and spoon excavators, with caries detector dye applied for identification. Hemostasis was achieved using cotton pellets and a solution of 5.25% sodium hypochlorite. Additionally, for radiographic evaluation, Kodak DF-57 films (Eastman Kodak Company, Rochester, New York, United States) were utilized and captured via the long-cone paralleling technique with X-mind DC x-ray equipment. These materials were crucial for conducting dPC procedures and assessing clinical and radiographic outcomes throughout the study period.

Data collection

The patient information form was utilized to record all pertinent data, such as demographic information, chief complaints, and medical and dental histories. The study involved the assignment of scores to the different clinical and radiological criteria based on their distinct characteristics. The outcomes of initial evaluations and subsequent follow-up assessments were recorded in the patient’s preoperative initial assessment sheet and recall assessment sheet, respectively. The operative data encompassed information such as the specific type of capping material used (either novel tricalcium silicate cement or calcium hydroxide), the date of the dPC procedure, the response to the cold test and electric pulp test, tooth mobility, probing pocket depths, and attachment loss, and the type and class of restoration placed following the pulp capping.

Clinical indicators

The clinical criteria used in this study included measuring pain on a visual analogue scale of 10, assessing percussion sensitivity, determining response to the cold and electric pulp tests, assessing tooth mobility, evaluating the presence or absence of an abscess, fistula, or sinus tract, measuring swelling of the periodontal tissues, and measuring any additional clinical symptoms of irreversible pulpitis, such as spontaneous pain. These assessments were made during both the initial and follow-up assessments.

Radiographic parameters

A radiologist utilized the long-cone paralleling technique (X-mind DC X-ray equipment, Olgiate Olivia, Italy) to capture periapical images. The study utilized Kodak DF-57 films that underwent development and fixing processes. The radiographic evaluation involved assessing the periradicular status of the chosen teeth using the PAI Scoring System (PAI Score). The standardized reading circumstances involved the use of a slide viewer with a magnification of 6.6X. The technique for examining the radiographs was standardized. For each of the five categories on the scale, scores were allocated using visual references. Following every follow-up appointment, a radiographic assessment was conducted to evaluate the periapical condition of the pulp-capped tooth. Subsequently, scores were allocated and recorded in the patient information form. During the recall examinations, the radiographic assessment was performed to detect any indications of periodontal ligament (PDL) space widening, alterations in the lamina dura, and external or internal root resorption related to the pulp-capped tooth.

Standard operating procedure

**Figure 1:**
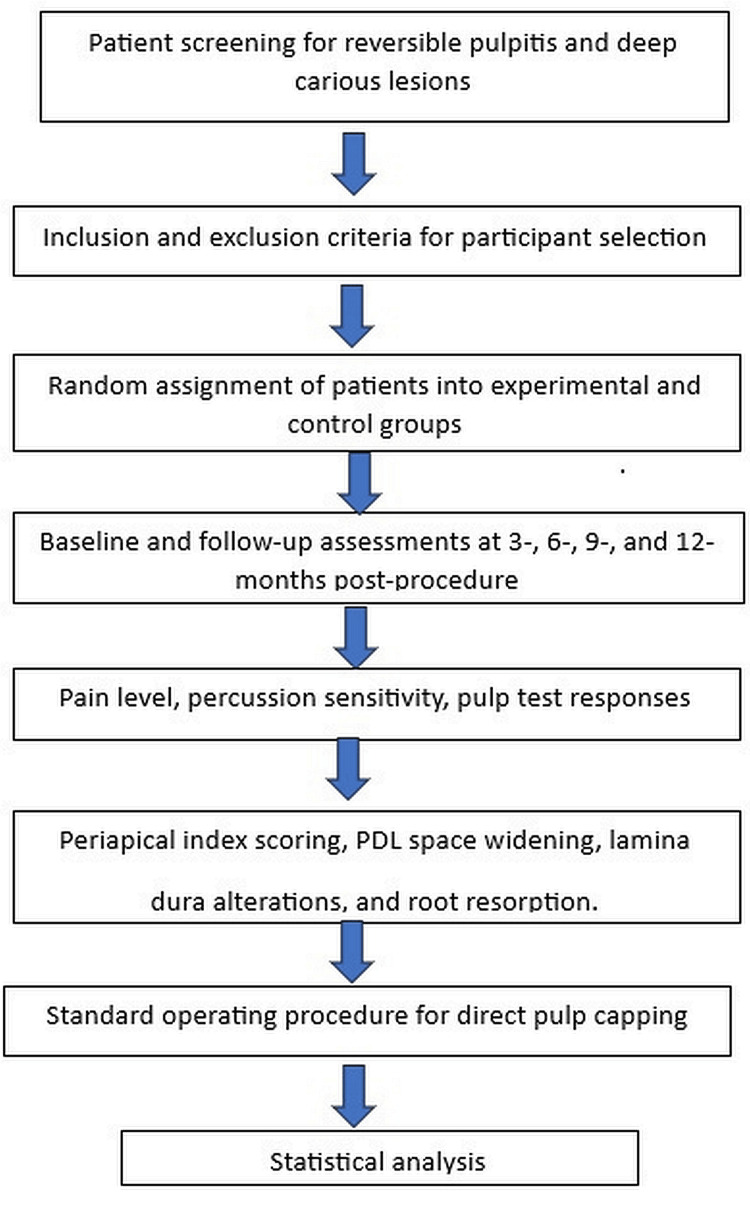
Flowchart of the methodology PDL, periodontal ligament

Following the administration of sufficient local anesthesia using Lignocaine HCl 2% with 1:80,000 adrenaline, a rubber dam was placed to isolate the tooth. The process of caries removal involves using mechanical excavation to remove the decayed tissue. Enamel that has been undermined is eliminated with a diamond bur, while soft debris is removed using a spoon excavator. Subsequently, the affected dentine was subjected to air drying, followed by the application of a caries detector dye for 10 seconds. The tooth was then rinsed and dried. The caries were completely removed using a slow-speed carbide round bur and spoon excavators until there was no or only minimal discoloration remaining. The staining method continued until it ceased to color the dentine. Prioritizing caution, efforts were made to eliminate the peripheral decay before excavating the caries from the cavity walls close to the pulp. However, one specific area of decay was removed, leading to the inadvertent exposure of the pulp. The last decayed area was eliminated, and the tooth was opened in a sterile manner using a no. ¼ round bur, resulting in an exposed site with a diameter of 0.5 mm. Hemostasis is accomplished by utilizing cotton pellets and a solution of 5.25% sodium hypochlorite, which is kept in place for around five minutes. This process not only aids in clearing the cavity but also disinfects it. When bleeding from the exposed pulp stops within five minutes, it suggests that the inflammation is reversible. In such cases, the exposed pulp is treated with either Biodentine or calcium hydroxide, depending on the sample group. The chosen agent is applied promptly over the exposed pulp tissue in a gentle manner without applying any pressure. If, during the process, it was noted that caries had deeply infiltrated the pulp chamber or if bleeding from the exposed pulp continued for five minutes or more, it was concluded that irreversible inflammation of the pulp tissue had occurred. Subsequently, dPC was not commenced, and instead, vital pulp extirpation was carried out, followed by the initiation of standard endodontic therapy. These teeth were not included in this study. Subsequent evaluations were conducted at intervals of three, six, nine, and 12 months after the procedure was performed (Figure 1).

Evaluation of outcomes

The outcome was evaluated on each subsequent assessment using clinical and radiographic characteristics. It was categorized as either successful or unsuccessful depending upon the scores received for each parameter.

Requirements for effective dPC

The clinical criteria for successful pulp capping include a definitive positive reaction to both cold and electric pulp tests, the lack of any clinical indications or symptoms of irreversible pulpitis, and the preservation of normal tooth function, such as no tooth mobility. Radiographic assessment was conducted using PAI scoring at each of the follow-up sessions and was deemed successful if PAI = 1, showing the presence of normal periapical structures.

Indicators for unsuccessful dPC

The outcome was deemed a failure if any of the following observations were made during any of the follow-up sessions concerning the treated tooth: The patient’s condition is characterized by various clinical manifestations, such as sensitivity to percussion or bite tests, pain, or discomfort associated with the capped tooth. Additionally, the patient exhibits a negative response to the cold test and the electric pulp test. Radiological examination reveals indications for apical periodontitis with a PAI score of 2 or higher. Furthermore, evidence of an abscess, sinus tract, or fistula is observed. Treatment options include root canal treatment or extraction of the pulp-capped tooth. Other complications that may arise include internal or external root resorption, as well as loss of tooth mobility.

Statistical analysis

The data was evaluated using IBM SPSS Statistics for Windows, Version 26.0 (Released 2019; IBM Corp., Armonk, NY, USA). The data is presented in terms of frequency and percentage, as well as measures of central tendency such as mean, median, and standard deviation. To better comprehend the comparisons and associations between various parameters, a t-test and a Chi-square (χ2) test were employed to contrast the average values between the two groups. A significance level of less than 0.05 was used for all statistical analyses.

## Results

The total sample size was divided into two groups: the control group, in which dPC was performed using calcium hydroxide (Dycal), and the case group, in which a novel tricalcium silicate cement (Biodentine) was utilized. Each group consisted of 30 patients. Among the 70 patients chosen, 10 patients (five patients in the control group and five patients in the case group) did not attend any of the subsequent assessments and were consequently excluded from this study. The control group consisted of 12 males and 18 females, while the Biodentine group had 13 males and 17 females. The age range of this group spanned from 16 to 34 years, with a mean age of 22.35 ± 2.3 years. The age and gender distributions were determined to have no statistically significant differences between the groups.

The pain score exhibited statistical significance over the three-month and six-month follow-up periods (p < 0.05). The pain score at the 12-month follow-up was 0.38 ± 0.52 in the case group and 0.42 ± 0.61 in the control group. The difference in pain scores between the two groups was not statistically significant (p = 0.79). The average PAI score for the Dycal and Biodentin groups after the 12-month follow-up was 1.38 ± 0.97 and 1 ± 0.3 (Table 1), respectively.

**Table 1:** Comparison of pain score and PAI score between the two groups at different follow-up periods Pain score: case group (0.38–1.22); control group (0.42–1.87) PAI score: case group (1.0–1.78); control group (1.38–2.23) PAI, Periapical Index

Variables		Three-month follow-up			Six-month follow-up			Nine-month follow-up			Twelve-month follow-up		
Parameter	Group	Mean + SD	t-test	p-value	Mean + SD	t-test	p-value	Mean + SD	t-test	p-value	Mean + SD	t-test	p-value
Pain score	Cases (n = 30)	1.22 ± 0.8	-3.27	0.002	0.85 ± 0.4	-2.17	0.03	0.43 ± 0.75	0.22	0.83	0.38 ± 0.52	-0.27	0.79
	Controls (n = 30)	1.87 ± 0.74			1.17 ± 0.7			0.47 ± 0.68			0.42 ± 0.61		
PAI score	Cases (n = 30)	1.78 ± 0.3	-3.67	0.001	1.71 ± 0.29	-2.83	0.006	1.04 ± 0.9	-3.63	0.001	1 ± 0.3	-2.05	0.044
	Controls (n = 30)	2.23 ± 0.6			2.12 ± 0.74			1.8 ± 0.71			1.38 ± 0.97		

This difference was found to be statistically significant (p = 0.044). In addition, there was a statistically significant difference in PAI score at the three-month, six-month, and nine-month follow-up visits (p < 0.01). During the entire follow-up period, one patient in the Biodentine group had discomfort upon percussion, a negative reaction for pulp vitality, pulpal alterations, and a widening of the periodontal space. The pulp vitality response was determined to be statistically insignificant across the groups during the entire assessment period (p > 0.05). Statistically significant differences in pulpal changes such as obliteration or narrowing and pain on percussion were noticed over the nine-month and 12-month follow-up periods (p < 0.05), as indicated in Table 2.

**Table 2:** Comparison of various parameters during the different follow-up periods between the two groups PDL, periodontal ligament

Variables		Three-month follow-up			Six-month follow-up			Nine-month follow-up			Twelve-month follow-up		
Parameter	Group	N (Positive/negative)	Chi-square test	p-value	N (Positive/negative)	Chi-square test	p-value	N (Positive/negative)	Chi-square test	p-value	N (Positive/negative)	Chi-square test	p-value
Percussion tenderness	Cases	29/1	1.96	0.16	28/2	0.74	0.39	29/1	4.04	0.04	29/1	4.04	0.04
	Controls	26/4			26/4			24/6			24/6		
Vitality	Cases	29/1	0.35	0.55	29/1	1.07	0.3	29/1	2.96	0.08	29/1	2.96	0.08
	Controls	28/2			27/3			25/5			25/5		
PDL space widening	Cases	1/29	1.07	0.3	1/29	1.96	0.16	1/29	1.96	0.16	1/29	4.04	0.04
	Controls	3/27			4/26			4/26			6/24		
Pulpal changes	Cases	29/1	0.35	0.55	29/1	1.07	0.3	29/1	4.04	0.04	29/1	5.19	0.02
	Controls	28/2			27/3			24/6			23/7		

**Figure 2:**
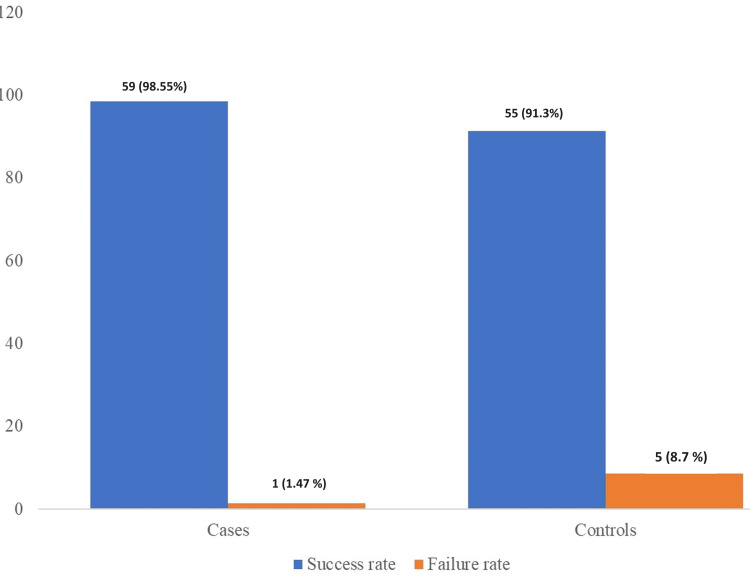
Comparison of the success rate of dPC in the study sample dPC, direct pulp capping

After 12 months, the success rate in the Dycal group was 55 (91.3%), and in the Biodentine group, it was 59 (98.55%). This difference was determined to be statistically significant (χ2 = 5.46; p = 0.019) (Figure 2).

**Figure 3:**
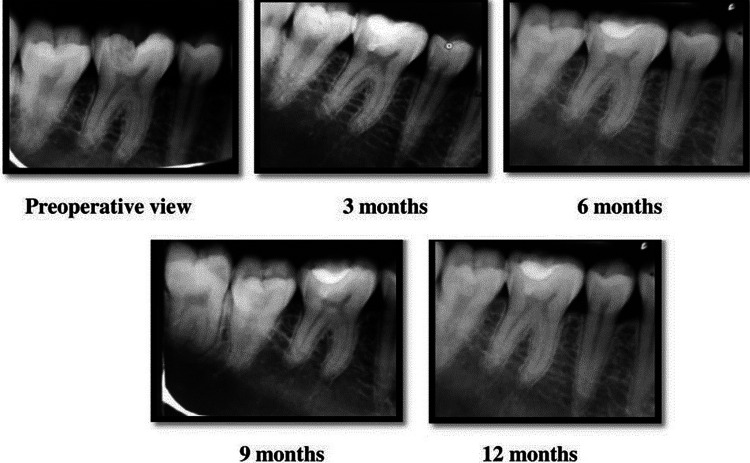
Radiographic evaluation of the periradicular status in the case (Biodentine) group during the study period

The radiological assessment done based on the PAI scores obtained during each of the follow-ups for the case group is illustrated in Figure 3.

**Figure 4:**
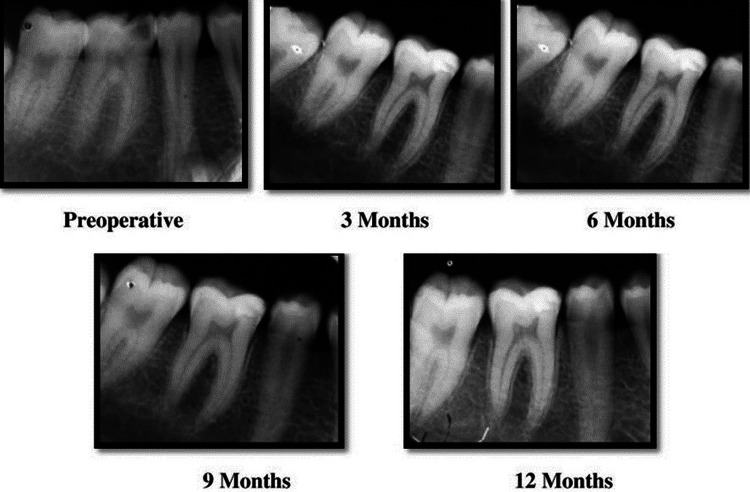
Radiographic evaluation of the periradicular status in the control (Dycal) group during the study period

Any evidence of internal or external root resorption, PDL space widening, or pulpal changes like obliteration or narrowing was noted. Radiographic follow-up examinations of periradicular structures were carried out at three months, six months, nine months, and 12 months postoperatively and are shown in Figure 4.

## Discussion

New insights into pulp physiology, caries advancement, mediators of inflammatory responses, and pulpal defense systems have altered the therapeutic paradigm for vital pulp therapies and the approach by which the treatment of caries is approached clinically. While numerous histological investigations have examined the effectiveness of the novel tricalcium silicate cement compared to calcium hydroxide in various dental procedures such as apexogenesis, apexification, root-end filling, and furcal perforation repair, there is currently a lack of prospective clinical research evaluating the efficacy of this cement as a dPC agent in teeth with deep carious lesions when compared to calcium hydroxide.

Biodentine cement contains calcium silicate and has mechanical qualities comparable to dentin. It can be employed as a substitute for dentin on crowns and roots, comparable to the way MTA is used [8-11]. It exerts a beneficial impact on vital pulp and promotes the development of tertiary dentin [11-13]. Reparative dentine development is further facilitated by its proximity to vital pulp. The liquid is made up of calcium chloride, which is combined with the superplasticizing reagent polycarboxylate to act as a water-reducing agent and setting accelerator in an aqueous form [8,9]. The powder comprises small hydrophilic granules that solidify when combined with the liquid. The process of adding water to the powder leads to the formation of a colloidal gel, which then transforms into a solid form within a time frame of around nine to 12 minutes. The mechanical strength gradually increases, surpassing 200 MPa within 24 hours and further improving over time, reaching a peak of 300 MPa within one month. The hardness of the material reaches 69 HVN over one month [8,9,12].

Biodentine does not have the disadvantages of calcium hydroxide, but it does have the same mechanism of action and indications. The main characteristics of this product are the advent of reactive dentin to preserve the vitality of the pulp, complete remineralization with the development of a calcific barrier, antimicrobial characteristics owing to an elevated pH level of 12.5, natural micro-mechanical anchorage for improved sealing with no need for surface treatment, comparable mechanical characteristics and behavior as human dentin, and an aluminum radiopacity of 3.5 mm, which allows for easy short- and long-term monitoring. Additionally, it can be used in a single visit as a base or alternative for dentin under permanent restorations such as composites for vital pulp therapy [14,15].

Laurent et al. evaluated the efficacy of Biodentine, in promoting the production of reparative dentine. They also examined its potential to regulate the production of TGF-β1 by pulp tissues. The study revealed that Biodentine stimulated the development of mineralized foci shortly after its use, manifested as osteodentine, and exhibited markers characteristic of odontoblasts. In addition, it became apparent that Biodentine had a substantial impact on the release of TGF-β1 from pulp tissues, regardless of an upsurge in the contact surface. The bioactive tricalcium silicate was found to have the capacity to stimulate the development of odontoblasts from pulp precursors, as observed by histology. The mineralized material that was obtained exhibited the molecular properties of dentin. Therefore, it indicates its potential application as a dPC agent [9]. Over extended periods, researchers identified the development of a uniform dentin bridge at the site of the lesion. This bridge was produced by cells exhibiting the characteristics of odontoblasts. On the other hand, the reparative tissue stimulated by Ca(OH)2 exhibited a porous framework, indicating a distinct reparative mechanism compared to those triggered by calcium silicate cement. Therefore, it can be inferred that the assessed cement is suitable for dPC [8].

Biodentine, which exhibits similar effectiveness to MTA, the present material of choice for reparative and regenerative operations, has numerous advantages over it. Biodentine has superior handling characteristics in comparison to MTA, resembling the consistency of zinc phosphate. In contrast, MTA has a granular texture that hinders effective compaction. Additionally, Biodentine has a regulated setting time of nine to 12 minutes, whereas MTA requires a lengthy two-hour duration for a complete setting. Therefore, it allows for the entire treatment to be completed in one session [7]. In contrast to other products based on Portland cement, this particular product is sufficiently durable to be utilized for both protecting pulp and filling temporary cavities [16]. Additionally, it is exceptionally economical.

According to Willershausen et al., the success rate of dPC with calcium hydroxide is 80.1% [17]. The study conducted by Al-Hiyasat et al. reported a rate of success of 33.3% for dPC using calcium hydroxide [18]. However, the high success rate seen in our study surpasses the outcome achieved by prior investigations (91.3%). Nevertheless, the success rate observed in our investigation was inferior to the findings of a long-term clinical trial conducted by Bogen et al. In their study, they obtained a success rate of 97.96% during a nine-year follow-up duration for dPC with MTA [19]. Due to recent advancements and breakthroughs in the field of material science, we have made significant progress in vital pulp therapy beyond the limited use of calcium hydroxide and its variants. The introduction of bioactive advancements in Portland types of cement has ushered in a new age in the arena of vital pulp therapies. This has transformed it into an effective therapy option with improved long-term effectiveness rates, as opposed to a temporary measure to maintain pulpal vitality until root canal treatment becomes imperative. Past research conducted by Barthel et al. on calcium hydroxide indicated a success rate ranging from 13% to 37% [20]. However, more recent experiments using Portland types of cement, such as those conducted by Strassler and Levin, have reported success rates of Biodentine as high as 95.3% [21]. These findings provide further evidence to support the present study findings.

The postoperative follow-up visits were conducted at three months, six months, nine months, and 12 months in the present investigation. A clinical investigation conducted by Matsuo et al. examined the results of dPC. The study indicated that three months was sufficient for a preliminary prognosis and to decide when to proceed with final restorations. This conclusion was based on the similarity in the rate of success, which varied from 80% to 83.3% observed in groups with postoperative intervals for follow-up that ranged from three to 18 months [22]. This indicates that the duration of the follow-up interval utilized in our investigation was adequate for assessing the results of dPC.

We utilized a solution of sodium hypochlorite with a concentration of 5.25% for both hemostasis and disinfection in our investigation. Previous research indicates that a 5.25% concentration of sodium hypochlorite has a solvent effect that is confined to the outer layer of pulp cells without affecting the underlying pulp tissue [23]. Another crucial aspect was the capacity to manage bleeding from the site of exposure. The study conducted by Matsuo et al. found that the extent of bleeding during pulp exposure had a substantial impact on the success rate of dPC. Specifically, the group with noticeable bleeding had a significantly reduced success rate compared to the group with minimal bleeding [22].

An underlying factor could be that the extent of bleeding at the point of exposure may serve as an indicator of the inflammatory condition of the pulp. If the inflammation of the pulp becomes more advanced, there is a considerable likelihood of developing irreversible pulpitis and experiencing significant bleeding upon exposure. A further potential cause is the existence of an extra-pulpal blood clot situated between the surface of the lesion and the pulp-capping material. Schroder et al. found that extra-pulpal blood hinders the healing process due to the presence of fibrin in the blood clot that can attract polymorphonuclear leukocytes, which function as a chemotactic component, leading to prolonged inflammation. He proposed that the presence of blood clots on the surface of the pulp could serve as a microbial substrate, drawing microorganisms to the point of exposure. For our investigation, we specifically chose teeth where bleeding at the point of exposure could be stopped within a time frame of three to five minutes [6].

Accurate delineation of success and failure is essential in a study aimed at documenting the rates of success and failure of a healthcare practice. Significant variations may arise in individual cohorts across various studies, encompassing factors such as patients’ age, oral health condition, and pulp pathology before the treatment. Additionally, the inconsistent criteria for determining success or failure provide considerable challenges when comparing the outcomes of different pulp-capping research. In this investigation, a successful outcome was precisely determined by the presence of entirely normal radiographic and clinical indications concerning the pulp-capped tooth. We failed to account for several factors, such as the reactions of different age groups and genders, the location of exposure, the specific tooth involved, the extent and quality of the restoration, and the effectiveness of the coronal restoration during each follow-up examination. According to Dammaschke, the crucial aspect of successfully capping the pulp is to prevent microorganisms from infiltrating. However, patient age and the magnitude or location of pulp exposure are at most of minor importance or entirely insignificant [7].

There are specific constraints on the investigation. Since the evaluation of success and failure was exclusively reliant on clinical and radiographic observations, it may not accurately correspond to the histologic state of the pulp after dPC. Therefore, it is advisable to conduct investigations that establish a correlation between the clinical and histomorphological observations after dPC. Additionally, conducting further studies with larger cohorts, employing more objective diagnostic assessments, and extending the duration of follow-up are imperative to establishing Biodentine as the preferred material for pulp capping.

Limitations of the study include potential biases related to the single-center design, which may limit the generalizability of the findings to a broader population. Additionally, the relatively small sample size could impact the statistical power of the study and increase the risk of type II errors, potentially leading to inaccurate conclusions. The follow-up period of 12 months might not be sufficient to capture longer-term outcomes or potential late complications associated with the different pulp capping materials. Furthermore, the study did not assess patient-reported outcomes or consider factors such as socioeconomic status or oral hygiene practices, which could influence treatment outcomes. Future research could address these limitations by conducting multicenter studies with larger sample sizes, longer follow-up periods, and comprehensive assessments of patient-reported outcomes and potential confounding variables.

## Conclusions

The study findings indicate that the new tricalcium silicate cement (Biodentine) outperforms calcium hydroxide in preserving the long-term health of the dental pulp after dPC. After the 12-month follow-up period, the Biodentine group achieved a total rate of success higher than the Dycal group.
